# Robust fiber orientation distribution function estimation using deep constrained spherical deconvolution for diffusion-weighted magnetic resonance imaging

**DOI:** 10.1117/1.JMI.11.1.014005

**Published:** 2024-01-05

**Authors:** Tianyuan Yao, Francois Rheault, Leon Y. Cai, Vishwesh Nath, Zuhayr Asad, Nancy Newlin, Can Cui, Ruining Deng, Karthik Ramadass, Andrea Shafer, Susan Resnick, Kurt Schilling, Bennett A. Landman, Yuankai Huo

**Affiliations:** a Vanderbilt University, Department of Computer Science, Nashville, Tennessee, United States; b Université de Sherbrooke, Department of Computer Science, Sherbrooke, Québec, Canada; c Vanderbilt University, Department of Biomedical Engineering, Nashville, Tennessee, United States; d NVIDIA Corporation, Bethesda, Maryland, United States; e National Institute on Aging, Laboratory of Behavioral Neuroscience, Baltimore, Maryland, United States; f Vanderbilt University, Department of Electrical and Computer Engineering, Nashville, Tennessee, United States

**Keywords:** diffusion magnetic resonance imaging, modeling, deep learning

## Abstract

**Purpose:**

Diffusion-weighted magnetic resonance imaging (DW-MRI) is a critical imaging method for capturing and modeling tissue microarchitecture at a millimeter scale. A common practice to model the measured DW-MRI signal is via fiber orientation distribution function (fODF). This function is the essential first step for the downstream tractography and connectivity analyses. With recent advantages in data sharing, large-scale multisite DW-MRI datasets are being made available for multisite studies. However, measurement variabilities (e.g., inter- and intrasite variability, hardware performance, and sequence design) are inevitable during the acquisition of DW-MRI. Most existing model-based methods [e.g., constrained spherical deconvolution (CSD)] and learning-based methods (e.g., deep learning) do not explicitly consider such variabilities in fODF modeling, which consequently leads to inferior performance on multisite and/or longitudinal diffusion studies.

**Approach:**

In this paper, we propose a data-driven deep CSD method to explicitly constrain the scan–rescan variabilities for a more reproducible and robust estimation of brain microstructure from repeated DW-MRI scans. Specifically, the proposed method introduces a three-dimensional volumetric scanner-invariant regularization scheme during the fODF estimation. We study the Human Connectome Project (HCP) young adults test–retest group as well as the MASiVar dataset (with inter- and intrasite scan/rescan data). The Baltimore Longitudinal Study of Aging dataset is employed for external validation.

**Results:**

From the experimental results, the proposed data-driven framework outperforms the existing benchmarks in repeated fODF estimation. By introducing the contrastive loss with scan/rescan data, the proposed method achieved a higher consistency while maintaining higher angular correlation coefficients with the CSD modeling. The proposed method is assessing the downstream connectivity analysis and shows increased performance in distinguishing subjects with different biomarkers.

**Conclusion:**

We propose a deep CSD method to explicitly reduce the scan–rescan variabilities, so as to model a more reproducible and robust brain microstructure from repeated DW-MRI scans. The plug-and-play design of the proposed approach is potentially applicable to a wider range of data harmonization problems in neuroimaging.

## 1 Introduction

Diffusion-weighted magnetic resonance imaging (DW-MRI) provides a noninvasive approach to estimate the intravoxel tissue microarchitectures as well as the reconstruction of *in vivo* neural pathways of the human brain.^1^ Reproducible fiber orientation distribution function (fODF) estimation is essential for downstream tractography and connectivity analyses.^2^ Recent advances in imaging technologies, such as high angular resolution diffusion imaging (HARDI),^3^ provide us with a higher angular resolution for modeling intravoxel orientation uncertainty. In contrast, diffusion tensor imaging,^4^ while useful, does not inherently provide this higher resolution due to its limitations in representing multiple fiber orientations within a single voxel. These new capabilities in imaging, particularly with HARDI, result in more precise depictions of white matter microstructure. However, they also necessitate more sophisticated processing methods due to the increased complexity of the data.

**Fig. 1:**
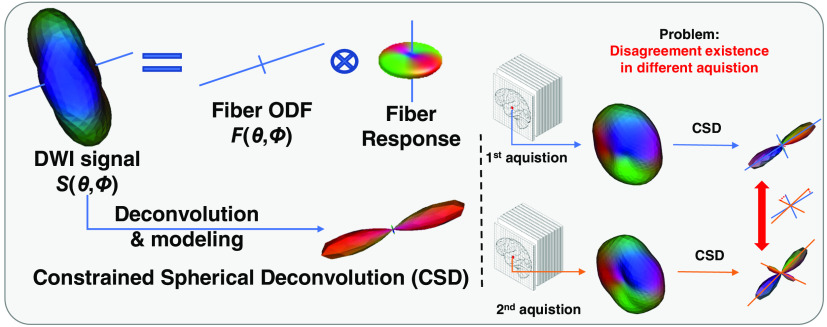
Overview of the proposed method. The CSD is affected by measurement factors of diffusion weighted imaging (DWI) signals (e.g., hardware, reconstruction algorithms, and acquisition parameters). The right panel shows the intersite variability during modeling, even for the data that are collected from the same patient.

The first family of the ODF estimation methods is typically called “modelbased,” which links underlying tissue microstructures with observed signals via sophisticated mathematical modelings such as constrained spherical deconvolution (CSD),^5^^,^^6^ Q-ball,^7^ and persistent angular structure.^8^ Among such approaches, CSD is one of the most broadly accepted for modeling HARDI signals.^9^ However, CSD is plagued by limited reproducibility (Fig. 1). Several studies have highlighted the biases, inaccuracies, as well as other limitations of HARDI methods in characterizing tissue microstructure (e.g., parameter selection, noise sensitivity, and assumptions).^9^ Moreover, such methods exhibit high computational complexity and often require a high number of acquisition points, which might not be available in clinical settings.^10^

To address such challenges, the second family of approaches—“data-driven” methods—is attracting increasingly more interest. For example, machine learning (ML) and deep learning (DL) techniques have demonstrated their remarkable abilities in neuroimaging.^10^^,^^11^ Such approaches have been applied to the task of microstructure estimation, aiming to directly learn the mapping between input DW-MRI scans and output fiber tractography. To maintain the necessary characteristics and reproducibility for clinical translation, robust training is carried out on a diverse and representative dataset in a data-driven method, ensuring the model can handle a variety of patient demographics and scanner variability. The trained model is subsequently validated and tested on independent datasets to evaluate performance and confirm its generalizability. The replicability of the model is assessed, with a thorough evaluation of its consistency across different test sets and scanners. By not assuming a specific diffusion model, data-driven algorithms can reduce the dependence on data acquisition schemes and additionally require less user intervention.

However, measurement variabilities (e.g., inter- and intrasite variability, hardware performance, and sequence design) are inevitable during the imaging process of DW-MRI (Fig. 1). Moreover, most existing model- and learning-based methods do not explicitly consider such variabilities in modeling, which consequently leads to inferior performance on multisite and/or longitudinal diffusion studies.^12^ To alleviate such issues, Nath et al.^13^ presented a multilayer perceptron (MLP)-based DL method for estimating discrete fODF from voxel-wise DW-MRI signals. In this work, Nath et al.^13^ incorporate identical dual networks to minimize the influence of scanner effects via scan–rescan data while learning the mapping between input DW-MRI signals and fODFs. The novelty in this approach is that paired data are used to drive the training of the network where it learns to ignore features of scanner noise and interscanner bias, which would otherwise lead the network to differentiate between the data.

Assessing scan–rescan consistency in DW-MRI studies is a common metric for validating the reproducibility of a proposed method because it directly tests the stability of the method under realistic conditions. Variability between multiple scans of the same subject could arise from a number of sources such as slight differences in patient positioning, physiological changes in the patient, or even minor fluctuations in scanner performance. A method that produces consistent results across repeated scans is likely to be more reliable and robust, enhancing its potential for clinical application. We followed this approach to ensure the modeling reproducibility in our study.

However, the three-dimensional (3D) context information was largely ignored in the “voxel-based” MLP model (i.e., model each voxel independently without considering the nearby voxels). It might lead to inferior performance for the 3D DW-MRI signals. To the best of our knowledge, this is the first data-driven approach to explicitly model the 3D patch-wise scan–rescan reproducibility for fODF estimation.

In this paper, we propose a novel deep CSD method to explicitly reduce the scan–rescan variabilities, so as to model a more reproducible and robust brain microstructure from repeated DW-MRI scans. Different from the voxel-wise learning in Nath et al.,^13^ we introduce a new volumetric patch-based modeling method for 3D DW-MRI signals. Briefly, the 3×3×3 3D patches from spherical harmonics (SHs)-represented DW-MRI signals are employed for a single-shell microstructure estimation. The deep convolutional neural network (CNN) is deployed as the computational model in our approach to derive the coefficients.

Another innovation is that we add intrasubject data augmentation to alleviate the impacts of a smaller number of diffusion directions on both reproducibility and the accuracy of metrics derived from CSD. The scan/rescan data are employed to facilitate our new loss function in reducing the intrasubject variability. The method has been trained, validated, and tested on both the Human Connectome Project (HCP) young adults (HCP-ya) test–retest group^14^ and the MASiVar dataset.^15^ To assess the model’s generalizability, we applied our model to the Baltimore Longitudinal Study of Aging (BLSA) dataset as the external validation.^16^ With both direct deployment and further finetuning, we witnessed an increasing consistency between intrasubject scans. Additionally, the brain structural connectomes are computed from the deep CSD as the downstream task for further model evaluation.

Our contribution is fivefold:

• We propose a novel deep CSD method to explicitly reduce the scan–rescan variabilities, to model a more reproducible and robust brain microstructure from repeated DW-MRI scans. To the best of our knowledge, this is the first data-driven approach to explicitly model the 3D patch-wise scan–rescan reproducibility for fODF estimation.
• Different from the previous voxel-wise learning studies, we introduce a new 3D volumetric representation of DW-MRI signals for a single-shell microstructure estimation.
• We propose a new intrasubject augmentation strategy that increases model robustness under the “fewer diffusion directions” scenarios.
• The ensuring of model reproducibility empowers better a predicative power in brain connectome analysis.
• The proposed method is a “plug-and-play” design as a simple multilayer regression network, which can be easily aggregated with downstream connectivity analysis.

## 2 Related Work

Ensuring reproducibility has been an important research topic in MR imaging, specifically in diffusion imaging.^17^^,^^18^ To control the site effect in multisite imaging data, several strategies have been proposed. Such approaches can be summarized into two major categories: conventional statistics-based method methods^17^^,^^19^^–^^23^ and more recent ML-based methods.^24^^–^^26^

### 2.1 Statistics-Based Methods

Conventional statistical methods are usually applied in a linear regression manner on univariate metrics with sites indexed as a categorical covariate, such as the least squares-based general linear model^19^ and Bayesian estimation-based ComBat.^17^^,^^20^ These methods have been utilized in multisite imaging studies and have shown a powerful capacity for removing linear site effects in brain metrics.^21^^–^^23^ However, noticeable limitations have been observed for this type of method. First, the site effect is mathematically assumed to be linear, whereas the actual effect can be fundamentally more complicated. Second, brain characteristics are considered independently in these models, largely neglecting the spatial and topological relationships among brain regions.

#### 2.1.1 ML-Based Approaches

Recently, proposed DL-based harmonization methods, including U-Net,^24^ cycle generative adversarial network,^27^ or 3D CNN,^28^ allow for mapping the complex abstract representations of the nonlinear spatial pattern of the site effects. These models have been primarily applied to ensure reproducibility of diffusion images,^29^ structural images,^25^ and morphological measurements,^26^ successfully eliminating the site effect with complex spatial or topological information. The model training strategy of site pairing is a common approach for DL-based methods. Briefly, the fusion of data from multiple sites from a single model greatly increases the generalizability of DL models. Most methods are ensuring test–retest reliability on the signal level.

As for tractography, most DL-based methods are dealing with the modeling process.^30^^–^^32^ By regarding different data as the gold standard, these approaches are chasing perfect fitting with different ground truth (GT) data, but the feasibility/confidence of the GT remains risky. For instance, Nath et al.^13^ used monkey histology data as GT for fiber orientation distributions (FODs). Such data are hard to obtain for a large human cohort, risking the generalizability of the trained model. Sedlar et al.^33^ regarded the human data from HCP as GT, which has over three shells with b-values of 1000, 2000, and $3000\;{s/\mathrm{mm}}^{2}$ (each with 90 gradient directions). However, such acquisition settings are not common in clinical settings. Additionally, even the same subject collected at two different settings may have some degrees apart. As a result, they might have very similar diffusion signals but fundamentally different fODFs. In our study, we proposed a robust fitting with GT while constraining the scan–rescan variabilities through modeling.

## 3 Method

### 3.1 Data Representation

SHs are functions defined on the sphere. A collection of SH can be used as a basis function to represent and reconstruct any function on the surface of a unit sphere.^34^ All diffusion signals are transformed to SH basis signal ODF as a unified input for DL models, using SHs with the “tournier07” basis.^35^ For the SH coefficients $c_{k}^{m}$, k is the order, m is the degree. For a given value of k, there are 2m+1 independent solutions of this form, one for each integer m with −k≤m≤k. In practice, a maximum order L is used to truncate the SH series. By only taking into account even-order SH functions, the above bases can be used to reconstruct symmetric spherical functions.

### 3.2 Architecture

**Fig. 2:**
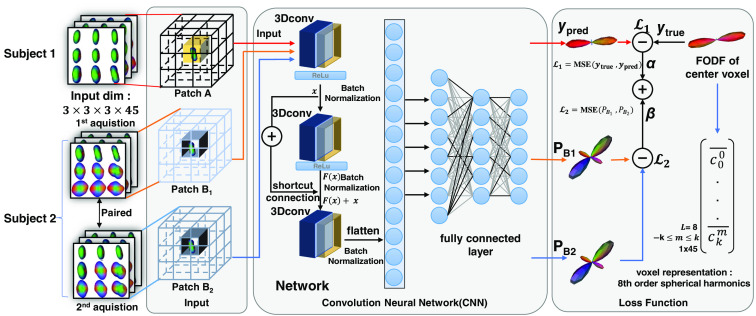
DL architecture. A 3D patch-wise CNN is proposed to fit the fODF from the SHs using 3×3×3 cubic DW-MRI signals. A contrastive loss is introduced to reduce the intrasubject variability.

Inspired by Nath et al.,^13^^,^^32^ we employ a 3D CNN with a residual block and utilize 3×3×3 cubic patches as inputs. The rationale is that 3D patches might provide more complete spatial information for DL networks (Fig. 2) compared with modeling each individual voxel independently. Eighth-order SH is used as data representation in our study. Briefly, the input size of the network is 3×3×3 with 45 channels, whereas the outputs are 45 eighth-order SH coefficients. During training, the architecture takes three patches as input. The first patch comes from one subject, where the network learns the direct mapping between DW-MRI signals and fODFs of the center voxel through a traditional loss (from the network output relative to a “truth” fODF). The remaining two are paired patches extracted from scan/rescan DW-MRI of another subject and the network learns to minimize the difference of the fODFs of the center voxel through a reproducibility loss. The same network is used for all three patches. The total loss is propagated back into the neural network to promote both estimation accuracy and reproducibility. For validation of the proposed method, it is in terms of accuracy relative to withheld voxels and reproducibility with paired voxels in scan–rescan imaging. The inclusion of scan/rescan data during training ensures the network’s robustness to input variations. However, in the inference stage, the model is designed to work with single patches, eliminating the need for paired data. The training process equips the model for real-world scenarios without reliance on a rescan image. The introduction of scan/rescan data is to make the network robust to variations in the input data.

In this network, three subsequent convolutional layers serve as the critical components of the CNN with 3D convolutional filters (conv1: 45 filters, 1×1 kernel size, padding=0; conv2: 45 filters, 3×3 kernel size, padding=1; and conv3: 45 filters, 3×3 kernel size, padding=0). One residual block is included to allow for the direct transmission of information from input and the third convolutional layer, thereby enabling the effective training of DL networks (shortcut connection). The convolutional filters are then flattened and connected to two dense layers for predicting fODF at the center voxel locations. All layers have rectified linear unit (ReLU) as the activation function. To enhance model training efficiency and stability, we employ batch normalization in our DL architecture to standardize the inputs to each layer of the network, reducing internal covariate shifts.

### 3.3 Loss Function

We introduce a customized loss function, as shown in Eqs. (13). The first term is the mean squared error (MSE) loss between the network’s FOD prediction and the GT with the hyperparameter “α.” N is the number of samples, m is the order of the SH basis, and $c_{k}^{m}$ is the SH coefficients. The second term is the MSE loss between a corresponding/paired of voxels (u and v). The second term has an expectation to be 0 and the hyperparameter is “β.” Specifically, if no scan/rescan data participate during training, “β” is set to 0

$$\begin{array}{c} \text{Loss}1=\frac{1}{N}\overset{N}{\underset{i=1}{\sum }}\overset{L}{\underset{k=0}{\sum }}\overset{k}{\underset{m=-k}{\sum }}{\left((c_{k}^{m}{)}_{\text{true},i}-(c_{k}^{m}{)}_{\mathrm{pred},i}\right)}^{2} \end{array},$$ (1)

$$\begin{array}{c} \text{Loss}2=\frac{1}{N}\overset{N}{\underset{i=1}{\sum }}\overset{L}{\underset{k=0}{\sum }}\overset{k}{\underset{m=-k}{\sum }}{\left((c_{k}^{m}{)}_{u,i}-(c_{k}^{m}{)}_{v,i}\right)}^{2} \end{array},$$ (2)

$$\begin{array}{c} \text{Loss}=\alpha *\text{loss}1+\beta *\text{loss}2. \end{array}$$ (3)

### 3.4 Intrasubject Data Augmentation

To provide a robust microstructure estimation, we introduce intrasubject data augmentation during our network training. By performing random diffusion directions dropout and feeding the model with augmented data during training, the model learns to handle situations such as missing or corrupt diffusion directions due to factors such as patient movement or hardware malfunction and make accurate predictions even with incomplete data. Furthermore, this augmentation strategy can improve the model’s generalizability by exposing it to a wider range of data scenarios, thus enabling it to better handle the variability and complexity inherent in real-world diffusion data. An additional b-vector check is performed to ensure that the rest of the directions are still well distributed on a sphere. Thus, we have reconstruction results from different total numbers of diffusion directions from the same DW-MRI signal. The CSD method is sensitive to the number of diffusion directions and therefore the generated fODF are augmented. By applying this augmentation, the diffusion signal ODF generated from fewer diffusion directions is labeled by the CSD results with the full numbers of gradient directions during the training process.

## 4 Experiments

The experiments can be summarized as with/without scan/rescan data, and with/without intrasubject augmentation on two DL models (voxel-wise MLP presented by Nath et al.^13^ and ours in Fig. 2). We assessed the models, as well as benchmarks, using the overall mean angular correlation coefficient (ACC) on white matter voxels between the prediction and the GT on the HCP and MASiVar dataset. The BLSA dataset has been introduced to further test model reproducibility.

We conducted studies to examine the robustness of the model with “fewer gradient directions.” The results are quantitatively evaluated with single-shell single-tissue CSD (ssst-CSD). Full direction ssst-CSD is regarded as the silver standard.

Eventually, complex network measures of brain structural connectomes (modularity, average betweenness centrality, characteristic path length, and global efficiency) are computed as an example of the downstream task evaluation.^36^

### 4.1 Data and Data Process

For the HCP-ya dataset,^14^ 45 subjects with the scan–rescan acquisition were used (a total of 90 images). A T1 volume of the same subject was used for white matter (WM) segmentation using 3D spatially localized atlas network tiles (SLANT).^37^ HCP was distortion corrected with topup and eddy.^38^^,^^39^ The acquisitions at b-value of 1000 and $2000\;{s/\mathrm{mm}}^{2}$ (each shell with 90 diffusion directions) were extracted for the study. Thirty subjects were used as training data, 10 were used for validation, and five were used for testing.

For the MASiVar dataset,^15^ five subjects were acquired on three different sites referred to as “A,” “B,” and “C.” Structural T1 was acquired for all subjects at all sites. All *in vivo* acquisitions were preprocessed with the PreQual pipeline^40^ and then registered pairwise per subject. The acquisitions at b-value of 1000 and $2000\;{s/\mathrm{mm}}^{2}$ (each shell with 96 diffusion directions) were extracted for the study. Two subjects from sets “A” and “B” were used as paired training data. One subject was used for validation, whereas two subjects were used for testing. Scans from site “C” were only used for evaluation.

Twenty-four subjects with the scan–rescan acquisition from the BLSA^16^ were used as an additional model evaluation cohort. The BLSA dataset was acquired at a b-value of $700\;{s/\mathrm{mm}}^{2}$ using a Philips 3T scanner. The data were preprocessed with PreQual. Eighteen subjects were used for training, two subjects were for validation, and four subjects were for testing. Additionally, another cohort of 198 subjects with biological information [age, sex, and ε4 allele of apolipoprotein E (APOE) states] was used for analyzing connectommes. Between them, 46 subjects were APOE positive while 152 subjects were APOE negative. They were preprocessed with the same protocol.

### 4.2 Model-Based Methods

We processed all the data to generate fODF with ssst-CSD using the diffusion imaging in Python (DIPY) library (version 1.50) with its default setting.^34^ Reconstructions from full diffusion direction DW-MRI were regarded as GT. Voxel-wise agreement (metrics; see Sec. 4.6) between paired data was calculated as silver standard.

### 4.3 ML-Based Methods

HCP and MASiVar datasets were employed to evaluate the learning-based method. The image data at a b-value of $2000\;{s/\mathrm{mm}}^{2}$ were used. We use a voxel-wise neural network as our DL baseline. The network consists of four fully connected layers. The number of neurons per layer is 400, 45, 200, and 45. The input is the 1×45 vector of the SH basis signal ODF, and the output is the 1×45 vector of the SH basis fODF. The architecture in Fig. 2 was used to evaluate the proposed patch-wise DL experiment.

Furthermore, the generalizability of different approaches was assessed by a rigorous external validation using BLSA dataset. The BLSA dataset was acquired at a b-value of $700\;{s/\mathrm{mm}}^{2}$. We used the image data at b-value of $1000\;{s/\mathrm{mm}}^{2}$ from both HCP and MASiVar to train the DL model using the same approaches in the previous experiments. Briefly, we trained the DL model with only BLSA data as baseline performance. To further test our model capacity, we further assess the scenarios of finetuning the last two linear layers, beyond applying our trained model on BLSA. Thus, we have three general approaches to compare the performance. Moreover, with/without scan/rescan data, and with/without intrasubject augmentation on two DL models (voxel-wise MLP presented by Nath et al.^13^ and our model in Fig. 2) are included as additional evaluation.

### 4.4 Ablation Study

In the ablation study, we evaluated the intrasubject consistency on all white matter voxels with a different number of diffusion directions. The DL model that had the best performance on the validation set is chosen for comparison. Different reconstruction results from a different number of diffusion directions were visualized as the qualitative results, whereas their agreements with the silver standard were assessed as the quantitative results.

Moreover, we investigated the impact of patch size on the performance of the patch-based method. We varied the patch size (3×3×3 and 5×5×5) and recorded the computational time and assessed the accuracy and reproducibility of the estimation. The input size of the first dense layer is adjusted due to the increased input size. The experiments were performed on a workstation with CPU: Intel Xeon Gold 6230R, 64GB memory, and GPU: Nvidia RTX A6000.

Additionally, we evaluated the effectiveness of the random dropout of diffusion directions using the MASiVar dataset. A subset of 45 diffusion directions from the same shell was first determined. A number of 45 was the basic requirement for eighth-order SHs and then visualized. Then the dropout (drops from 96 to the subset) was performed randomly 10 times. With both results of CSD reconstruction and DL prediction on the DW-MRI signal, the ACC and the mean diffusivity (MD) (zeroth-order SH of the fODF) were computed. The spheres in both single fiber and crossing fiber areas were presented to assess the effectiveness of the intrasubject augmentation by examining shapeshift.

### 4.5 Downstream Task Evaluation

To evaluate the performance on downstream tasks, we predicted APOE status using different brain structure connectome maps via different fODF modeling methods. Complex network measures of brain structural connectomes (modularity, average betweenness centrality, characteristic path length, and global efficiency) were computed from the results as the downstream task.^36^ During the process, fODFs were computed from both CSD and our DL networks. We used the MRTrix (version 3.0.3) default probabilistic tracking algorithm of second-order integration over FODs for tractography.^41^ We generated 10 million streamlines to build each tractogram, seeding, and termination using the five-tissue-type mask. We allowed backtracking. After, we converted the tractography to a connectome with the Desikan–Killany atlas^42^ with 84 cortical parcellations from Freesurfer.^43^ Graph theory measures were computed with the Brain Connectivity Toolbox (version 2019-03-03).^36^

Modularity was the degree to which the network may be subdivided into clearly delineated and nonoverlapping groups.^36^ Betweenness centrality was the fraction of the shortest paths in the network that contained a given node.^36^ Average betweenness centrality was the average fraction of shortest paths that nodes in a network participate in Ref. 36. The characteristic path length was the average shortest path between nodes in millimeters. Global efficiency was the average inverse shortest path length.^36^ Using the graph measures and biological information (age and sex) as input, we used a three-layer MLP network to perform classification across different APOE groups between CSD and the DL approaches.

We performed leave-one-out cross-validation as the evaluation strategy. Weighted cross-entropy was applied as we had unbalanced groups. The cross-validation had been performed 20 times, and the 95% confidence interval of the metrics was evaluated to alleviate the effect of random seeds.

### 4.6 Evaluation Metrics

To evaluate the prediction accuracy and responsibility of the proposed DL methods, we used the ACC [Eq. (4)] to evaluate the similarity of the prediction when compared with the GT estimate of CSD and consistency on scan/rescan images. ACC was a generalized measure for all fiber population scenarios. It assessed the correlation of all directions over a SH expansion. It is calculated between fODF of two voxels (u and v), where $u_{km}$ and $v_{km}$ are the SH coefficients. In brief, it provided an estimate of how closely a pair of fODFs was related on a scale of −1 to 1, where 1 was the best measure. Here, “u” and “v” represented sets of SH coefficients

$$\begin{array}{c} \mathrm{ACC}=\frac{\overset{L}{\underset{k=1}{\sum }}\overset{k}{\underset{m=-k}{\sum }}(u_{km})(v_{km}^{*})}{{\left[\overset{L}{\underset{k=1}{\sum }}\overset{k}{\underset{m=-k}{\sum }}{\left|u_{km}\right|}^{2}\right]}^{0.5}\cdot {\left[\overset{L}{\underset{k=1}{\sum }}\overset{k}{\underset{m=-k}{\sum }}{\left|v_{km}\right|}^{2}\right]}^{0.5}} \end{array}.$$ (4)

To robustly validate the significance and consistency of model performances, a deeper statistical assessment was necessary. For this purpose, we employed the Wilcoxon signed-rank test. This nonparametric test was chosen to compare paired differences without making assumptions about the normality of the distribution, making it ideal for our study. By using the Wilcoxon signed-rank test, we aimed to understand whether the observed differences in our model’s predictions were statistically significant or could have potentially arisen by random chance.

For downstream task evaluation, we used the classical classification metrics (accuracy, precision, recall, and F1) to evaluate the biomarker prediction. Macro precision, recall, and F1 were used in our study as we had unbalanced positive/negative APOE groups.

## 5 Results

### 5.1 DL Results

The results of the fODF estimation are presented in Table 1. The mean ACC over white matter regions is shown in the table to evaluate the similarity of the prediction when compared with the truth estimate of CSD. This serves as the accuracy of prediction. The mean ACC over white matter regions is calculated in the test cohort of scan/rescan imaging, shown as scan/rescan consistency in the table. This serves as validation of reproducibility.

**Table 1:** Performance of fODF prediction on HCP and MASiVar.

Model and method	Scan/rescan	Intrasubject augmentation	ACC, compare with full direction CSD	Scan/rescan consistency	
CSD (silver standard)	N/A	N/A	1	0.826	Ref
Voxel-wise			0.942	0.830	**★**
	✓		0.938	0.878	**★**
	✓	✓	0.939	0.882	**★**
Patch-wise			0.949	0.834	**★**
	✓		**0.954**	0.886	**★**
	✓	✓	0.953	**0.891**	**★**

Note: Mean ACC is calculated over white matter region voxels between (1) prediction and GT signals, (2) prediction of paired scan/rescan DWI signals are calculated. Bold values indicate the best performance in each reported metric. In the statistical assessment, we compared our model’s fODF prediction with the reconstruction from the silver standard and the ACC of the scan–rescan test cohort in HCP and MASiVar as compared with the silver standard. The results yielded a p-value of <0.001 (marked as **★**).

* Mean ACC are calculated over white matter voxels.

The implementation of the CNN network for 3D-patch inputs led to a superior SH coefficients estimation by incorporating more information from neighboring voxels. Meanwhile, by introducing the identity loss with scan/rescan data, the proposed method achieved a higher consistency while maintaining higher ACCs with CSD.

### 5.2 Model Evaluation on Unseen Dataset

By applying our method on BLSA dataset, as shown in Table 2, it shows a great improvement in scan/rescan consistency while applying our approaches and using BLSA as finetuning data as compared with both the silver standard and the model trained directly on the BLSA dataset (0.838 versus 0.834 versus 0.635). More importantly, by directly applying the model to unseen data, we still show significant intrasubject consistency and maintain high agreement (0.836 versus 0.872) with the full direction CSD.

**Table 2:** Performance on BLSA dataset.

Model and method	Training data	Test data	Scan/rescan	Intrasubject augmentation	ACC, compare with full direction CSD	Scan/rescan consistency	
CSD (silver standard)	N/A	BLSA	N/A	N/A	1 (upper bound)	0.635	Ref
Voxel-wise DL (Nath et al.^13^)	HCP and MASiVar	BLSA			0.845	0.747	**★**
	HCP and MASiVar	BLSA	✓		0.832	0.813	**★**
Patch-wise DL	HCP and MASiVar	BLSA			0.829	0.763	**★**
	HCP and MASiVar	BLSA	✓		0.834	0.812	**★**
	HCP and MASiVar	BLSA	✓	✓	0.836	0.824	**★**
	BLSA	BLSA	✓		**0.872**	0.834	**★**
	HCP, MASiVar, and BLSA (finetune)	BLSA	✓		0.849	0.828	**★**
	HCP, MASiVar, and BLSA (finetune)	BLSA	✓	✓	0.842	**0.838**	**★**

Note: The performance of different methods on the BLSA dataset. Bold values indicate the best performance in each reported metric. In the statistical assessment, we compared different model’s fODF prediction with the reconstruction from the silver standard and the ACC of the scan–rescan test cohort in BLSA as compared with the silver standard. The results yielded a p-value of <0.001 (marked as **★**).

### 5.3 Ablation Study

In the ablation study on the impact of patch size (Table 3), we applied the model- and DL-based methods to the test image and recorded the computational time required by each method. The results indicated that our DL-based method is faster than the voxel-based method, demonstrating the efficiency of the DL approach. The results also showed that while the estimation performance (both accuracy and reproducibility) with 5×5×5 patches was slightly better than with 3×3×3 patches, the computational time increased remarkably. Given this trade-off between estimation performance and computational efficiency, 3×3×3 patches were selected as the default setting across studies to balance accuracy and computational time effectively.

**Table 3:** Ablation study on the impact of patch size.

Model and method	ACC, compare with full direction CSD	Scan/rescan consistency	Computational time (s)
CSD (silver standard)	1	0.826	532.42
Voxel-wise	0.939	0.882	75.29
Patch-wise (3×3×3)	0.953	0.891	89.36
Patch-wise (5×5×5)	0.950	0.898	179.55

Note: All DL models are trained with scan/rescan data and the intrasubject augmentation strategy. For the accuracy and reproducibility of fODF estimation, an average performance on the test cohort of HCP and MASiVar is reported. For computational time, an average inference time on HCP subjects of size 145×174×145 is reported.

**Fig. 3:**
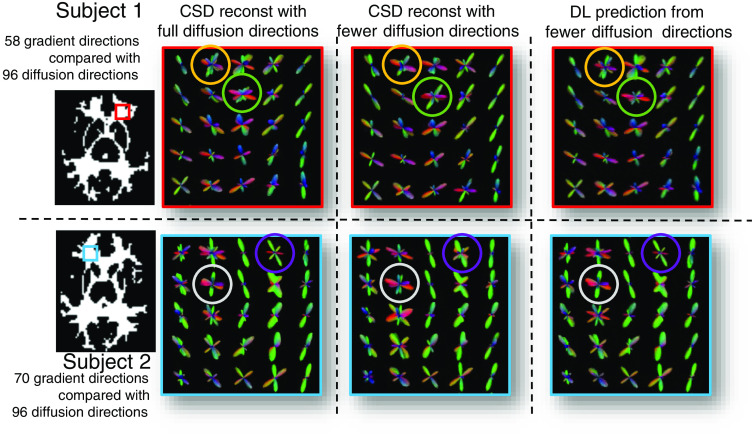
Qualitative results of fODF modeling. Visualizations of fODF of the proposed DL method and the results from CSD modeling on two testing subjects in MASiVar. We took the same patch (matched with the same color of the border) from the results in the crossing fiber area. The same voxel is matched with the same color of the circle.

**Fig. 4:**
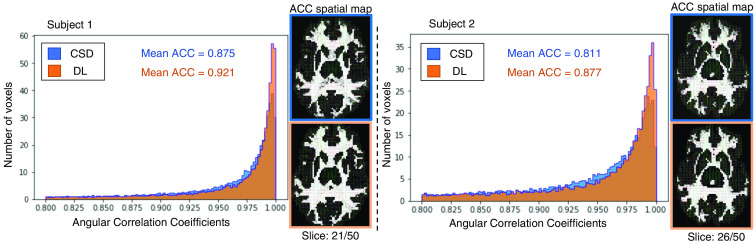
ACC histogram and ACC spatial map. This figure depicts the histogram of ACC between full diffusion directions’ reconstruction and fewer directions’ reconstruction while using the proposed DL method and the results from CSD modeling on the two testing subjects in MASiVar. The ACC spatial maps are the comparison between (1) the fODFs of reconstruction from CSD with full diffusion directions and (2) fewer diffusion directions’ CSD and DL estimator on two testing subjects.

In Table 4, we evaluate the intrasubject augmentation by comparing the intrasubject consistency on all white matter voxels with different numbers of diffusion directions. The DL model that has the best performance on the validation set is chosen for comparison. In Fig. 3, the right side shows a qualitative result of the visualization of the estimated SH coefficients, and the left side shows the comparison with full-direction CSD. By performing CSD, the test subjects with a mean of 72 diffusion directions can only maintain a mean ACC of 0.848 as compared with their same acquisition with 96 directions. By adding the intrasubject augmentation during the training process, both voxel-wise and patch-wise models have significant improvement, which shows that DL reveals untapped information during the ODF estimation. Figure 4 shows the result of (1) estimation on a signal with fewer diffusion directions using a patch-wise DL model with scan/rescan data and intra-subject augmentation participated during training and (2) CSD reconstruction result.

**Table 4:** Performance in fewer diffusion direction situation.

Model and method	Scan/rescan	Intrasubject augmentation	Intrasubject consistency	
CSD	N/A	N/A	0.848 ± 0.189	Ref
Voxel-wise			0.838 ± 0.195	**★**
	✓		0.849 ± 0.175	**★**
	✓	✓	0.879 ± 0.138	**★**
Patch-wise			0.842 ± 0.185	**★**
	✓		0.856 ± 0.173	**★**
	✓	✓	**0.902 ± 0.128**	**★**

Note: Model/method degradation is evaluated by assessing the prediction consistency with fewer diffusion direction signals. Bold values indicate the best performance keeping intrasubject consistency. Wilcoxon signed-rank test is applied as a statistical assessment. In the statistical assessment, we compared our model’s fODF prediction of the scan–rescan test cohort in MASiVar as compared with the silver standard. The results yielded a p-value of <0.001 (marked as **★**).

**Fig. 5:**
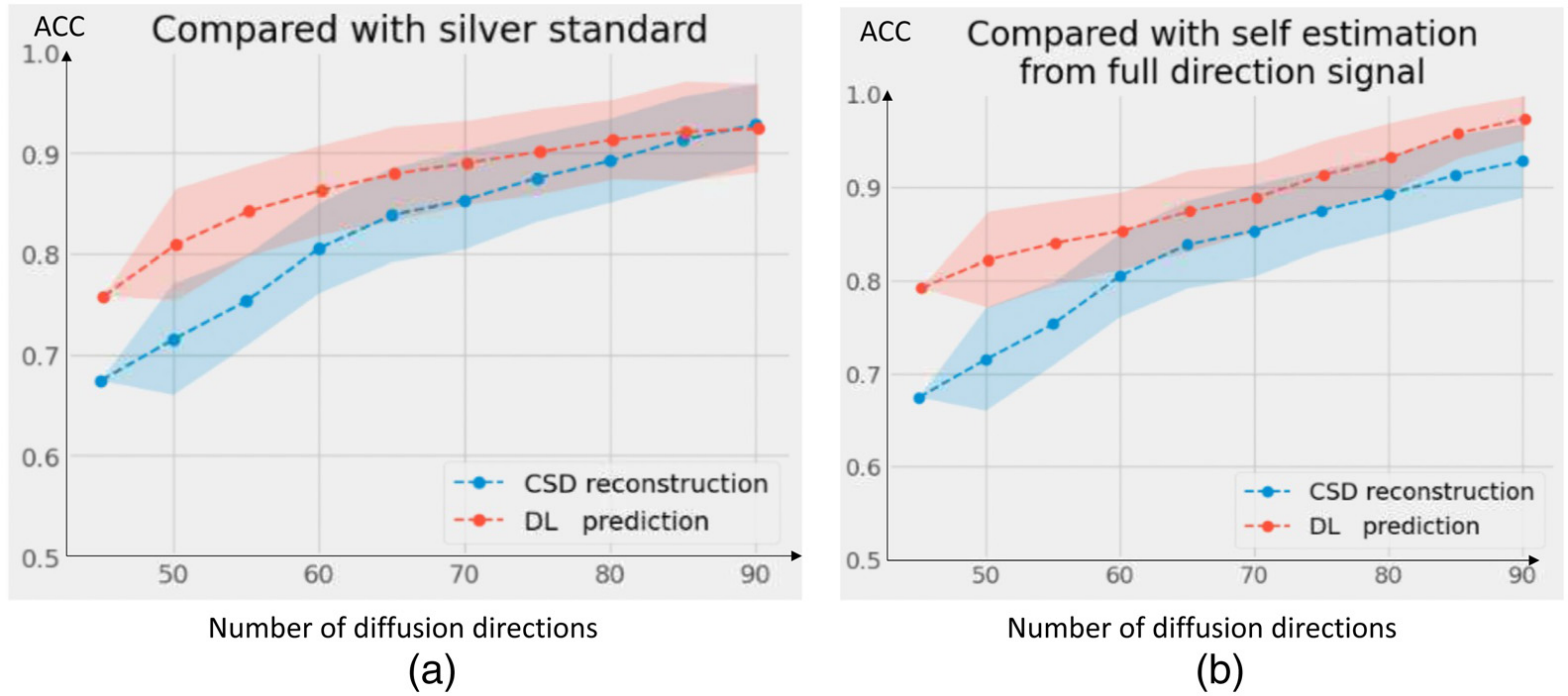
Quantitative result of performances of methods/modeling with diffusion direction dropout. ACC is calculated at specific intervals—every five diffusion directions—such as at 45, 50, 55, and so on, enabling us to evaluate how the consistency of the model’s/method’s output was preserved despite the reduction in diffusion gradient directions. The dropout (drops from 96 to the subset of 45 directions) was performed randomly 10 times. The mean ACC and the std are calculated and shown in the line chart. (a) The DL-based method maintains high consistency than the CSD reconstruction when both are compared with the silver standard (full-direction CSD) during the diffusion direction dropout. (b) A similar assessment, except comparing itself using the full-direction modeling.

We are evaluating our DL model by testing its performance with degraded signals, which involve artificially reducing input data quality (diffusion direction dropout). This assessment allows us to understand the model’s robustness under less ideal conditions by comparing its results with CSD. Figure 5 shows how different methods maintain consistency in their result compared with silver standard and methods’ self-full direction-estimation when faced with degraded input data.

**Fig. 6:**
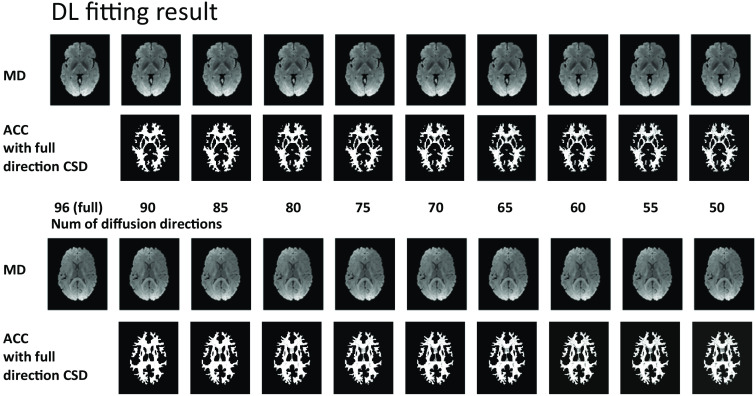
Qualitative visualization of the predicted coefficients in fewer diffusion directions scenario. The mean diffusivity map (MD, zeroth-order of the SH coefficient) and ACC agreement spatial map (even orders of the SH coefficient without zeroth-order, compared with the silver standard–full direction CSD) of DL results of the two MASiVar test subjects. The dropout was performed from 96 to the subset of 45 directions while the visualization was shown at intervals of every five diffusion directions.

**Fig. 7:**
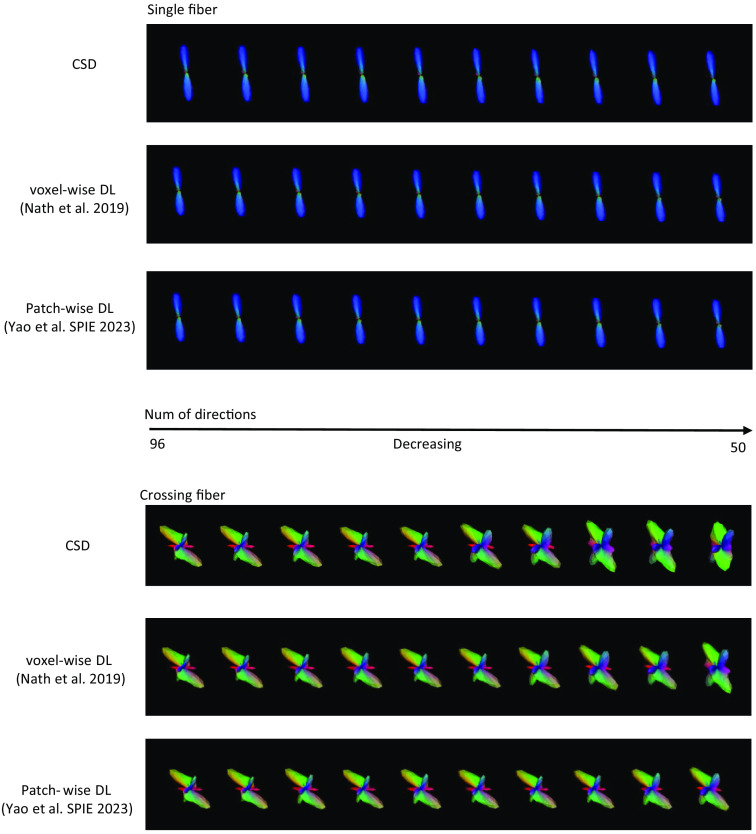
Qualitative results of synthetic spheres. Visualization of reconstruction from different numbers of diffusion directions of voxel from single/crossing fiber population. The dropout was performed from 96 to the subset of 45 directions while the visualization was shown at intervals of every five diffusion directions. CSD reconstruction of a voxel in crossing fiber population has an obvious shapeshift in the low-resolution scheme while DL with the data augmentation strategies remains a robust estimation.

Figures 6 and 7 provide an additional qualitative visualization of the robust DL fitting during diffusion direction dropout. In Fig. 6, we plot the mean diffusivity and the ACC spatial map where MD indicates the fitting of the zeroth-order coefficients (no. 1 in 45) and the ACC focuses on the rest of the SH coefficients (nos. 2 to 45 in 45). In Fig. 7, we focus on two unique voxels belonging to the single fiber population and crossing fiber population respectively, the result shows that CSD reconstruction has an obvious shapeshift in the low-resolution scheme while DL with the data augmentation strategies remains higher consistency, especially in the visualization of the crossing-fiber voxel.

### 5.4 Downstream Task Evaluation

The APOE states are predicted using different brain structure connectome maps. This task is employed as a downstream task to evaluate the performance of different fODF modeling methods. Briefly, we calculated the accuracy, precision, recall, and F1 for each method via 20 bootstraps. Then, the 95% confidence intervals (95% CIs) are calculated for all metrics to demonstrate the variability. As shown in Table 5, all lower bounds of DL metrics are higher than the upper bounds of the CSD method.

**Table 5:** Confidence interval on metrics of biomarker predictions.

Metrics	DL	95% CI		CSD	95% CI		p-value
		Lower bound	Upper bound		Lower bound	Upper bound	
Accuracy	0.568	[0.560	0.577]	0.526	[0.517	0.535]	<0.001
Precision	0.520	[0.513	0.528]	0.463	[0.456	0.472]	<0.001
Recall	0.525	[0.515	0.534]	0.450	[0.438	0.461]	<0.001
F1	0.511	[0.502	0.520]	0.441	[0.432	0.480]	<0.001

Note: The mean and the range of the 95% CI for the DL metrics are reported. The p-values for accuracy, precision, recall, and F1 score when comparing DL to CSD are <0.001, respectively. Indicating that the differences in these metrics between the two methods are statistically significant.

## 6 Discussion

Ensuring scan–rescan variability is of paramount importance in diffusion signal modeling, particularly when employing a data-driven approach. Data-driven methods, which learn directly from the data, inherently rely on the consistency and reliability of the input data to produce accurate and robust models. As such, the issue of scan–rescan variability comes into sharp focus. In the context of diffusion signal modeling, variations between repeated scans of the same subject can introduce inconsistencies that could significantly affect the performance of the data-driven models. This is because these models are sensitive to the statistical properties of the training data. If the input data are inconsistent due to scan–rescan variability, the learned model may not generalize well, leading to less accurate predictions. Thus, in our study, addressing scan–rescan variability is not just a quality control issue, but a crucial factor that directly impacts the reliability and clinical applicability of data-driven diffusion signal models. A consistent focus on minimizing this variability can result in models that provide more accurate, reliable, and clinically meaningful results.

In our study, we developed a data-driven fODF modeling algorithm to provide robust microstructure estimation for modeling tractography. The proposed method (1) learns the mapping from SH basis 3D DW-MRI signal to a fiber ODF, (2) improves consistency and alleviates the effects that occur between different scanners with a new loss function, (3) increases model robustness in the ‘fewer diffusion directions’ scenarios, and (4) empowers better a predicative power in downstream tasks (e.g., APOE states estimation from connectomes).

The improvement from voxel-wise input to patch-wise input is based on the assumption that the model is trained on a diverse patch selection, it sees a wide range of scenarios. And meanwhile, a DL model can capture the complex relationships as a deeper network is able to account for the 3D context without being unduly influenced by neighboring voxels in some scenarios. This is the advantage of data-driven method, we do not need to explicitly ensure that neighboring voxels have similar distribution functions to central voxel. Regarding the result of CSD from high-quality data as silver standard, the DL algorithm was designed to be robust to variations and noise in the data. By introducing specific loss functions and regularization techniques, the DL model can be better suited to handle variations in the testing data compared to the CSD algorithm. Additionally, the reproducibility loss functions can guide the model to focus on certain aspects (ensuring scan/rescan variability) of the data that might be overlooked by the CSD algorithm.

However, there are still several limitations in our approaches. First, one key limitation of our approaches is the cost of computing resources by performing patch-to-center predictions. We need to generate 27 times storage for the 3×3×3 patches for one single DW-MRI. Second, to provide more precise microstructure estimation, we need to target our model to multitissue multishell CSD, which provides different fiber response functions to different tissues. It also leads to a potential future question on how to encode the b-value information into SH representation during the transform of multishell DWI and signal ODFS.

## 7 Conclusion

In this paper, we propose a deep CSD method to explicitly reduce the scan–rescan variabilities so as to model a more reproducible and robust brain microstructure of repeated DW-MRI scans that are acquired from the same patient. From the experimental results, the proposed data-driven framework outperforms the existing benchmarks in fODF estimation. In general, our study is a step toward the direct harmonization of the estimated microstructure (e.g., FOD) using DL and a data-driven scheme when scan–rescan data are available for training. The proposed method is potentially applicable to a wider range of data harmonization problems in neuroimaging.

## Data Availability

Code can be given upon request. All datasets are freely available and unrestricted for non-commercial research purposes. These include the HCP-ya test–retest group^14^ (https://www.humanconnectome.org/study/hcp-young-adult), the MASiVar dataset^15^ (https://openneuro.org/datasets/ds003416), and the BLSA dataset^16^ (apply at https://www.blsa.nih.gov/).
